# GIP_HUMAN [22–51] Peptide Encoded by the Glucose-Dependent Insulinotropic Polypeptide (GIP) Gene Suppresses Insulin Expression and Secretion in INS-1E Cells and Rat Pancreatic Islets

**DOI:** 10.3390/genes14101910

**Published:** 2023-10-05

**Authors:** Emily Pusch, Małgorzata Krążek, Tatiana Wojciechowicz, Maciej Sassek, Paweł A. Kołodziejski, Mathias Z. Strowski, Krzysztof W. Nowak, Marek Skrzypski

**Affiliations:** 1 Department of Animal Physiology, Biochemistry and Biostructure, Poznan University of Life Sciences, 60-637 Poznan, Poland; emily.pusch@onet.eu (E.P.); malgorzata.krazek@up.poznan.pl (M.K.); tatiana.wojciechowicz@up.poznan.pl (T.W.); maciej.sassek@up.poznan.pl (M.S.); pawel.kolodziejski@up.poznan.pl (P.A.K.); kwnowak@up.poznan.pl (K.W.N.); 2 Department of Hepatology and Gastroenterology, Charité-University Medicine Berlin, 13353 Berlin, Germany; mathias.strowski@charite.de; 3 Medical Clinic III, 15236 Frankfurt, Germany

**Keywords:** beta cell, GIP, GIP_HUMAN [22–51], insulin, INS-1E, pancreatic islet, expression

## Abstract

GIP_HUMAN [22–51] is a recently discovered peptide that shares the same precursor molecule with glucose-dependent insulinotropic polypeptide (GIP). In vivo, chronic infusion of GIP_HUMAN [22–51] in ApoE−/− mice enhanced the development of aortic atherosclerotic lesions and upregulated inflammatory and proatherogenic proteins. In the present study, we evaluate the effects of GIP_HUMAN [22–51] on insulin mRNA expression and secretion in insulin-producing INS-1E cells and isolated rat pancreatic islets. Furthermore, we characterize the influence of GIP_HUMAN [22–51] on cell proliferation and death and on Nf-kB nuclear translocation. Rat insulin-producing INS-1E cells and pancreatic islets, isolated from male Wistar rats, were used in this study. Gene expression was evaluated using real-time PCR. Cell proliferation was studied using a BrdU incorporation assay. Cell death was quantified by evaluating histone-complexed DNA fragments. Insulin secretion was determined using an ELISA test. Nf-kB nuclear translocation was detected using immunofluorescence. GIP_HUMAN [22–51] suppressed insulin (*Ins1* and *Ins2*) in INS-1E cells and pancreatic islets. Moreover, GIP_HUMAN [22–51] promoted the translocation of NF-κB from cytoplasm to the nucleus. In the presence of a pharmacological inhibitor of NF-κB, GIP_HUMAN [22–51] was unable to suppress *Ins2* mRNA expression. Moreover, GIP_HUMAN [22–51] downregulated insulin secretion at low (2.8 mmol/L) but not high (16.7 mmol/L) glucose concentration. By contrast, GIP_HUMAN [22–51] failed to affect cell proliferation and apoptosis. We conclude that GIP_HUMAN [22–51] suppresses insulin expression and secretion in pancreatic β cells without affecting β cell proliferation or apoptosis. Notably, the effects of GIP_HUMAN [22–51] on insulin secretion are glucose-dependent.

## 1. Introduction

Blood glucose levels are regulated by numerous endocrine factors. Two major pancreatic hormones, insulin and glucagon, maintain glucose homeostasis through the complex regulation of glucose synthesis, intracellular glucose uptake and metabolism [1]. It is well-known that insulin deficiency and the loss of insulin-producing pancreatic β cell mass is a hallmark of type 1 diabetes [2]; however, this phenomenon has also been reported in type 2 diabetic patients [3]. Growing evidence indicates that pancreatic β cell functions, including the production and secretion of insulin, are modulated by a group of hormones termed incretins. It was found that incretins, such as glucagon-like peptide 1 (GLP-1) or glucose-dependent insulinotropic polypeptide (also termed gastric inhibitory polypeptide (GIP)), stimulate postprandial insulin secretion and β cell proliferation while attenuating β cell death [4,5,6]. Therefore, it is unsurprising that these hormones are extensively studied in the context of pathophysiology, therapy, and diabetic complications [7]. Recently, Masaki et al., when searching for new peptides that may potentially contribute to the modulation of cardiovascular system functions, identified the new polypeptide termed GIP_HUMAN [22–51], which shares a common precursor molecule with GIP [8]. GIP_HUMAN [22–51] is located on the N-terminal fragment of pre-pro-GIP peptide [8]. A healthy human tissue microarray stained with anti-GIP_HUMAN [22–51] revealed that high levels of GIP_HUMAN [22–51] were detected in the cerebellum, liver, stomach, kidney, heart, small intestine, and colon. Furthermore, lower levels of GIP_HUMAN [22–51] were found in the cerebral cortex, tonsils, lymph nodes, and male gonads [8]. Moreover, it was shown that GIP_HUMAN [22–51] did not colocalize with GIP at the cellular level. Furthermore, it is important to note that GIP_HUMAN [22–51] is present in the circulation in humans [8].

Studies on the biological role of GIP_HUMAN [22–51] showed that continuous infusion of GIP_HUMAN [22–51] for four weeks in mice potentiated the development of aortic atherosclerotic lesions and atheromatous plaque formation and increased proatherogenic proteins in an ApoE−/− mouse model. In contrast, infusion of the neutralizing GIP_HUMAN antibody blocked the effects on plaque formation. Therefore, the authors of this study suggested that GIP_HUMAN [22–51] can be considered as a new proatherogenic peptide [8].

It is important to note that abnormalities in cardiovascular systems are common complications in patients with type 1 and 2 diabetes [9,10]. GIP was implicated in the context of regulation of β cell physiology [6]; however, little is known about the role of GIP_HUMAN [22–51]. In the current study, we therefore evaluate the effects of GIP_HUMAN [22–51] on pancreatic β cell functions. We study the influence of this peptide on insulin expression and secretion as well as on β cell proliferation and cell death.

## 2. Materials and Methods

### 2.1. Materials

GIP_HUMAN [22–51] [8] was synthesized by Novazym (Poznań, Poland). INS-1E cells were purchased from AddexBio (San Diego, CA, USA). The NF-κB Activation Inhibitor IV was from Contact Santa Cruz Biotechnology, Inc. (Dallas, TX, USA). Other reagents were from Sigma-Aldrich (St. Louis, MO, USA), unless otherwise stated.

### 2.2. Cell Culture

Rat insulin-producing INS-1E cells (a well-established β cell model to study insulin secretion and β cell biology [11]) were cultured in appropriate conditions in the incubator (37 °C, 5% CO_2_, humid atmosphere). Cells were cultured in a RPMI 1640 medium supplemented with 10% fetal calf serum FCS, 2 mmol/L glutamine, 100 kU/L penicillin, 100 mg/L streptomycin, 10 mmol/L HEPES buffer, 1 mmol/L sodium pyruvate, and 50 μmol/L β-mercaptoethanol.

### 2.3. Isolation of Pancreatic Islets

Pancreatic islets were isolated from male Wistar rats weighing 300–350 g, as we previously described [12]. Animals were obtained from the Mossakowski Medical Research Centre Polish Academy of Sciences (Warsaw, Poland). Rats were housed under standard conditions (12/12 h light/dark cycle, 21 ± 1 °C). In brief, to isolate pancreatic islets, pancreases were injected with 8 mL of ice-cold Hank’s balanced salt solution (HBSS) containing 25 mmol/L HEPES and collagenase type XI (0.35 mg/mL). Then, excised pancreases were digested for 30 min in a water bath (37 °C). After digestion, isolated islets were purified with Histopaque 1077 discontinuous gradient. Purified islets were repeatedly washed with HBSS containing bovine serum albumin (0.2%) (Biowest, Nuaillé, France). All procedures were in accordance with the regulations and the ethical standards set by the National Ethics Commission for Investigations on Animals in Poland according to The Act on The Protection of Animals used for Scientific or Educational Purposes in Poland adopted on 15 January 2015 and to earlier regulations. Experiments where the analysis of tissues obtained from dead animals did not undergo any experimental procedures did not require any permission from the Local Ethical Commission for Investigation on Animals.

### 2.4. Real-Time PCR

After seeding, cells (3.2 × 10^5^ cells/well) were cultured in 6-well plates. After 24 h, the standard incubation medium was replaced by a serum-free medium, containing 0.1% free fatty acid and BSA, and the cells were incubated for an additional 3 h. Thereafter, GIP_HUMAN [22–51] peptide was added at a concentration of 0, 10 or 100 nmmol/L, and cells were then incubated for 3 and 24 h. The RNA was isolated using Extrazol (Blirt, Gdańsk, Poland). The quality and quantity of isolated RNA were determined using a NanoDrop 1000 spectrophotometer (Thermo Fisher Scientific, Waltham, MA, USA). RNA was stored at −86 °C for further analysis. Next, the RNA (0.5 µg) was reverse transcribed to cDNA using a FIREScript RT cDNA Synthesis KIT (Solis BioDyne, Tartu, Estonia). The cDNA obtained in the previous step was diluted 10-fold. The PCR reaction mix was prepared using specific primers and HOT FIREPol EvaGreen qPCR Mix Plus (Solis BioDyne, Tatru, Estonia). A PCR reaction was performed using QuantStudio 12 K flex (ThermoFosher Scientific). To calculate relative gene expression, the *Hprt1* reference gene was used. PCR product specificity was confirmed using melting curve analysis. The following sequences of primers were used: *Hprt1* (NM_012583.2): cagtcaacgggggacataa (left), ttggggctgtactgcttga (right), *Ins1* (NM_019129.3): gaggctctgtacctggtgtg (left), ccaaggtctgaagatccccg (right), *Ins2* (NM_019130.2): gtgaccagctacagtcggaa (left), accacaaaggtgctgtttgac (right), *Pdx1* (NM_022852.4): cctttcccgaatggaaccga (left), and ttttccacgcgtgagctttg (right).

### 2.5. Immunofluorescence Detection of NF-κB Nuclear Translocation

Nuclear translocation of Nf-kB was determined using immunofluorescence staining [13]. INS-1E cells were seeded on a Lab-Tek II glass-based culture Chamber Slide™ System (Nunc, Thermo Scientific, Waltham, MA, USA) at a density of 3.2 × 10^5^ cells/mL. After 24 h of preculture, cells were treated with 100 nmol/l GIP_HUMAN [22–51] for 3 or 24 h. Next, cells were fixed with 4% paraformaldehyde in PBS for 10 min at RT. After washing (3× ice-cold PBS), cell membranes were permeabilized using 0.2% Triton X-100 in PBS (10 min, RT) and washed (3 × 5 min, PBS). Non-specific bounds were neutralized (1% BSA, 10% rabbit serum in PBS, 1 h, RT) and incubated with a primary antibody (NF-kB p65/RelA Rabbit pAb (cat. no. A2547), Woburn, MA, USA). This antibody (diluted 1:160) was added for overnight incubation at 4 °C. PBS-Tween washing was performed twice, and membranes were incubated with a secondary antibody (Secondary Antibody Alexa Fluor^®^ 488 conjugate, A11008/Invitrogen, Thermo Scientific, Waltham, MA, USA) for 1.5 h at RT. After being washed three times for 3 min each at RT, cells were mounted using a Fluoroshield with DAPI (Sigma, St. Louis, MO, USA). Fluorescence was measured using a 488 nm excitation filter, and emissions were observed using a 520 nm emission filter under a LSM 510 inverted microscope (Carl Zeiss, Oberkochen, Germany). Images were processed using Axio vision v. 4.6 software.

### 2.6. Insulin Secretion

INS-1E cells were seeded in 24-well plates (8 × 10^4^ cells/well) and cultured for 24 h. Then, cells were washed with a PBS buffer, and a 500 μL Krebs glucose-free solution was added (NaCl 136 mmol/L, KCl 4.7 mmol/L, CaCl_2_ 1 mmol/L, MgSO_4_ 1.2 mmol/L, KH_2_PO_4_ 1.2 mmol/L, NaHCO_3_ 2 mmol/L, HEPES 10 mmol/L, pH 7.4). Incubation was conducted for 1 h. The role of GIP_HUMAN [22–51] peptide (10 mmol/L or 100 nmol/L) in insulin secretion was studied at low (2.8 mmol/L) or high (16.7 mmol/L) glucose concentrations. After incubation for 1 h, the KRB buffer was collected and centrifuged (200× *g* for 5 min) and samples were stored at −20 °C for the determination of released insulin. Insulin secretion was measured using rat insulin ELISA (Mercodia, Uppsala, Sweden). Insulin secretion was normalized to protein content, which was measured using a Pierce™ BCA Protein Assay Kit (ThermoFisher Scientific, Waltham, MA, USA). Insulin secretion from isolated pancreatic islets was determined, as previously described [12]. The absorbances were measured at 450 nm using a Synergy 2 Multi-Mode Microplate Reader (BioTek, Winooski, VT, USA).

### 2.7. Determination of Intracellular Insulin Content

INS-1E cells (1.6 × 10^5^ cells/well) were cultured in 12-well plates for 12 h in a normal growing medium. Next, the standard incubation medium was replaced by a serum-free medium containing 0.1% free fatty acid BSA, and the cells were incubated for an additional 3 h. Thereafter, GIP_HUMAN [22–51] peptide was added at a concentration of 0 or 100 nmol/L, and INS-1E cells were then incubated for 24 h. After the incubation, cells were washed with PBS, 1 mL of 75% ethanol containing 1.5% HCl was added, and cells were incubated overnight at −20 °C, as previously described [14]. After the incubation, the mixture of ethanol and HCl was collected and centrifuged at 250× *g*. Supernatant was collected and used to determine the intracellular insulin content using ELISA.

### 2.8. Cell Proliferation

First, INS-1E cells were cultured in 96-well plates (2 × 10^4^ cells/well). After 24 h, the incubation medium was exchanged for a serum-free medium, and cells were cultured for an additional 24 h at 37 °C. Afterwards, this medium was replaced by the medium supplemented with GIP_HUMAN [22–51] at 1, 10, or 100 nmol/L. Cells were then cultured for 24 and 48 h. The BrdU incorporation was tested using the Cell Proliferation ELISA BrdU (colorimetric) Kit (Roche Diagnostics, Manheim, Germany). In brief, after the incubation with the GIP_HUMAN [22–51] peptide, a BrdU solution with a final concentration of 10 µmol/L was added to each well, and cells were cultured in a cell culture incubator for an additional 2 h. Next, the cell culture medium was removed, 200 µL of FixDenat solution was added, and cells were incubated for 30 min at RT. Afterwards, the FixDenat solution was removed and 100 µL of Anti-BrdU-POD working solution was added. After the incubation (70 min at RT), plates were washed three times using the washing buffer. Next, a substrate solution (100 µL) was added, and plates were incubated for approximately 10 min. The reaction was stopped by the addition of 25 µL HCl. The absorbances of samples were measured at 450 nm (reference wavelength was 690 nm) using a Synergy 2 Multi-Mode Microplate Reader (BioTek, Winooski, VT, USA).

### 2.9. Cell Death

Apoptosis was measured by the Cell Death Detection ELISA PLUS Kit (Roche Diagnostics), which is suitable for determining cytoplasmic histone-associated DNA fragments (mono- and oligo-nucleosomes) during cell death. Before the test, cells at a density of 8 × 10^4^ cells per well (24 well-plates) were cultured in a serum-free medium for 3 h. Afterwards, a new medium supplemented with GIP_HUMAN [22–51] at 10 or 100 nmol/L concentration was added. Then, cells were incubated for 48 h in the presence or absence (control) of GIP_HUMAN [22–51]. Thereafter, cells were lysed in the Lysis Buffer, and apoptotic cells were measured using the Cell Death Detection ELISA PLUS kit according to the manufacturer’s protocol. Absorbances of samples at 405 nm (reference wavelength: 690 nm) were evaluated using the Synergy 2 Multi-Mode Microplate Reader (BioTek).

### 2.10. Statistical Analysis

Results are presented as the mean ± standard error of the mean. Statistical analyses were performed using GraphPad Prism version 8.0 (GraphPad Software, Inc., San Diego, CA, USA). One-way ANOVA followed by the Tukey post hoc test or if the normality test failed, non-parametric Kruskal–Wallis test were used. *p* < 0.05 (*) was considered as statistically significant difference.

## 3. Results

### 3.1. GIP_HUMAN [22–51] Modulates Insulin mRNA in INS-1E Cells and Isolated Rat Pancreatic Islets

**Figure 1:**
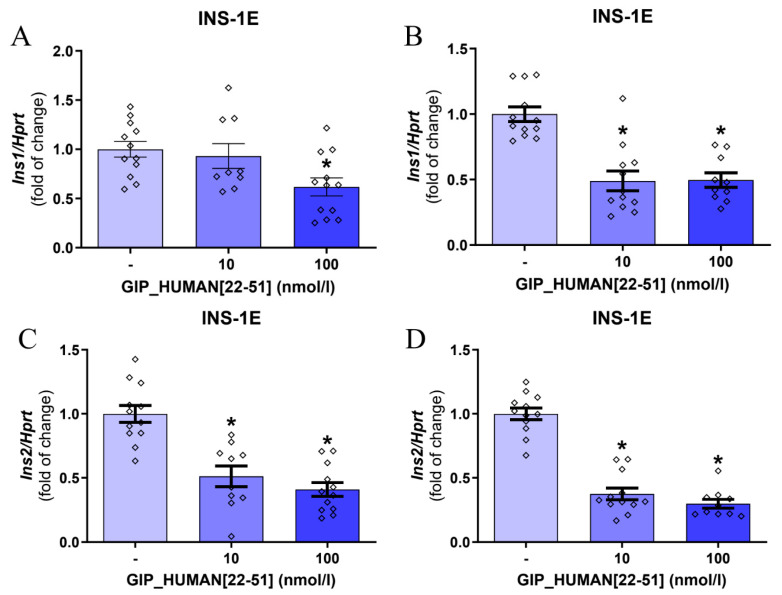

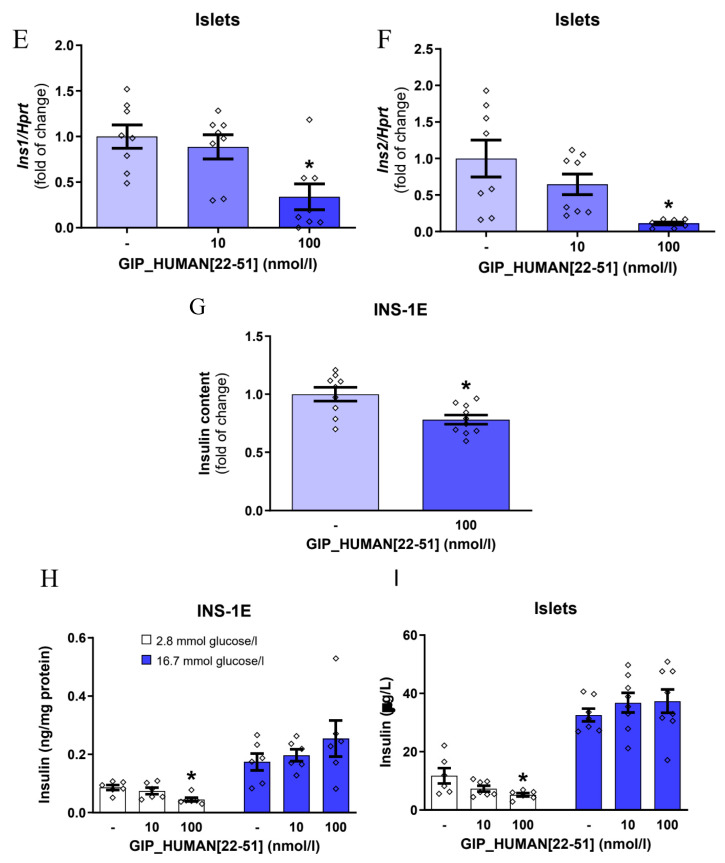
Effects of GIP_HUMAN [22–51] on insulin mRNA expression in INS-1E cells and isolated rat pancreatic islets. Expression of *Ins1* and *Ins2* in INS-1E cells exposed to GIP_HUMAN [22–51] for 3 (**A**,**C**) and 24 h (**B**,**D**). Expression of *Ins1* (**E**) and *Ins2* (**F**) in rat pancreatic islets treated with GIP_HUMAN [22–51] for 24 h. (**G**) Intracellular insulin content in INS-1E cells treated with (100 nmol/L) or without GIP_HUMAN [22–51] for 24 h. Insulin secretion assessed in INS-1E cells (**H**) and isolated rat pancreatic islets (**I**) treated with or without GIP_HUMAN [22–51] in the presence of 2.8 or 16.7 mmol/L glucose for 60 min. Results are mean ± SEM, (*n* = 9–12, (**A**–**D**), *n* = 7–8, (**E**,**F**), *n* = 9–10, (**G**), *n* = 6–8, (**H**,**I**)). * indicates *p* < 0.05 (compared to the control group).

Initially, we evaluated the effects of GIP_HUMAN [22–51] on insulin mRNA expression in INS-1E cells. Of note, rodents express two nonallelic genes of insulin (*Ins1* and *Ins2*). *Ins2* is an ortholog to the human insulin gene [15]. As shown in Figure 1, GIP_HUMAN [22–51] suppressed insulin 1 (*Ins1*) mRNA expression in INS-1E cells after 3 h (Figure 1A) and 24 h (Figure 1B). Moreover, GIP_HUMAN [22–51] downregulated *Ins2* mRNA expression assessed after 3 h or 24 h (Figure 1C,D). Additionally, the suppression of *Ins1* and *Ins2* mRNA expression was found in freshly isolated rat pancreatic islets exposed to GIP_HUMAN [22–51] at 100 nmol/L for 24 h (Figure 1E,F). Furthermore, to study whether the changes in expression of mRNA levels are accompanied by changes in protein production, we assessed the effects of GIP_HUMAN [22–51] on intracellular insulin content in INS-1E cells. As shown in Figure 1G, GIP_HUMAN [22–51] downregulated intracellular insulin content at the concentration of 100 nmol/L after 24 h.

Insulin secretion is precisely modulated by changes in blood glucose concentration and insulin sensitivity [16]. Therefore, we next studied whether GIP_HUMAN [22–51] modulates insulin secretion from INS-1E or rat pancreatic islets at the presence of low (2.8 mmol/L) or high (16.8 mmol/L) glucose concentrations [17]. As shown in Figure 1H,I, GIP_HUMAN [22–51] (100 nmol/L) suppressed insulin secretion from INS-1E cells and isolated rat pancreatic islets at low (2.8 mmol/L) but not high (16.7 mmol/L) glucose concentrations. In summary, these results indicate that GIP_HUMAN [22–51] suppresses insulin expression as well as insulin secretion at low glucose concentrations.

### 3.2. GIP_HUMAN [22–51] Activates NF-kB in INS-1E Cells

**Figure 2:**
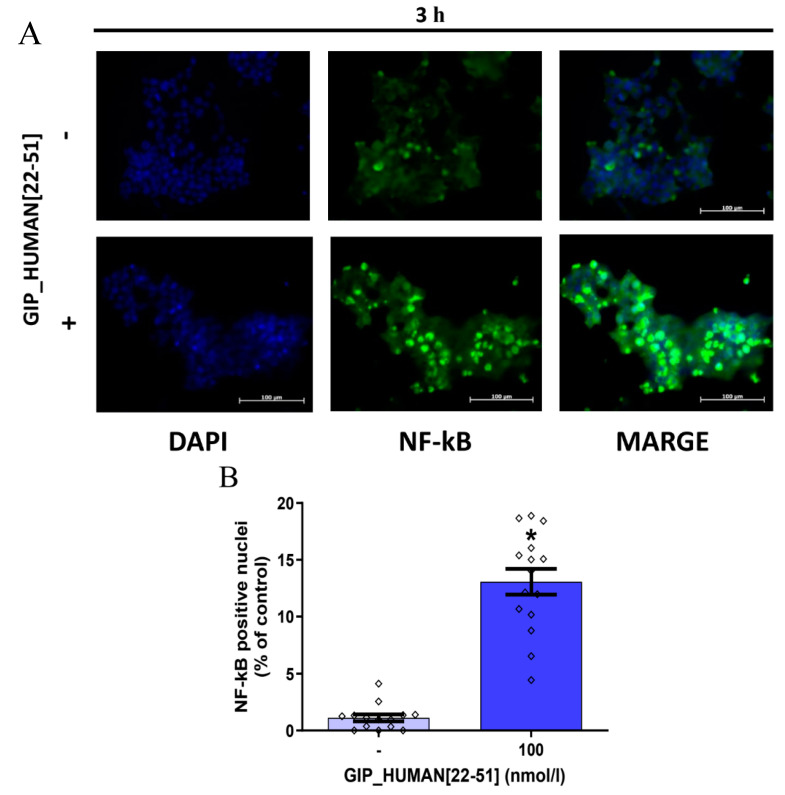

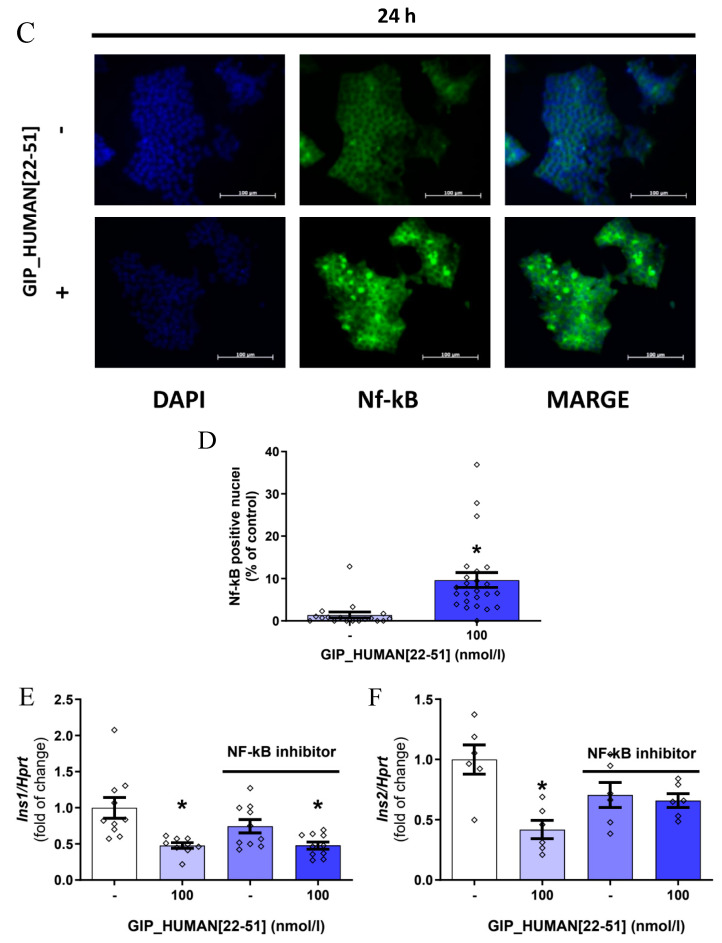
Effects of GIP_HUMAN [22–51] on nuclear translocation of Nf-kB in INS-1E cells. Immunofluorescence detection of Nf-kB in INS-1E cells treated with or without GIP_HUMAN [22–51] for 3 (**A**) or 24 h (**C**). The percentage of Nf-kB-positive nuclei in INS-1E cells treated with or without GIP_HUMAN [22–51] for 3 (**B**) or 24 h (**D**). NF-κB-positive and NF-κB-negative nuclei in each group were counted in at least 2500 cells from at least three independent experiments. (**E**,**F**) Effects of Nf-kB inhibitor on GIP_HUMAN [22–51] suppressed *Ins1* (**E**) (*n* = 9–10) and *Ins2* (**F**) (*n* = 6) mRNA expression. Results are mean ± SEM. * indicates *p* < 0.05.

It was reported that GIP_HUMAN [22–51] stimulates nuclear translocation of NF-κB in human vascular endothelial cells and macrophages [8]. Since there is evidence that NF-κB is implicated in controlling insulin mRNA expression in pancreatic β cells [18], we studied the effect of GIP_HUMAN [22–51] on NF-κB nuclear translocation in INS-1E cells. As demonstrated in Figure 2A–D, 100 nmol/l of GIP_HUMAN [22–51] caused translocation of NF-κB from cytoplasm to the nucleus after 3 h and 24 h, respectively. Furthermore, as shown in Figure 2E,F, NF-κB inhibitor IV attenuated GIP_HUMAN [22–51] suppressed *Ins2* mRNA expression but not *Ins1*.

### 3.3. GIP_HUMAN [22–51] Does Not Modulate Cell Proliferation and Death in INS-1E Cells

**Figure 3:**
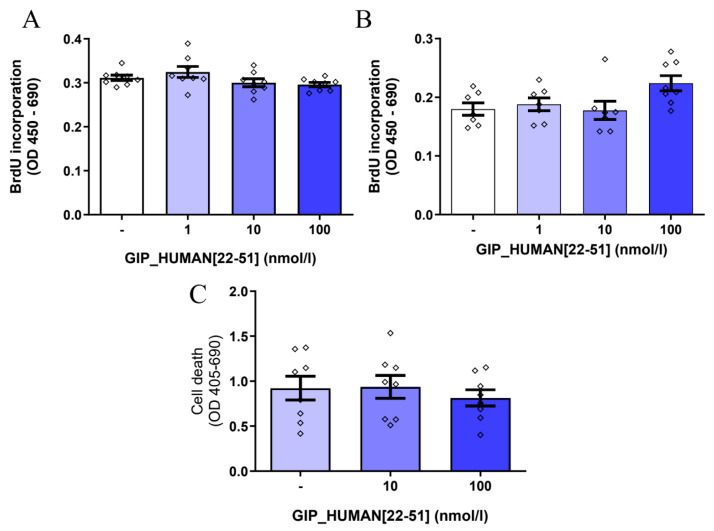
Effects of GIP_HUMAN [22–51] on INS-1E cell proliferation and apoptotic cell death. Cell proliferation determined in INS-1E cells treated with GIP_HUMAN [22–51] for 24 (**A**) or 48 h (**B**). (**C**) Effects of GIP_HUMAN [22–51] on cell death after 48 h. Results are mean ± SEM, (*n* = 7–8).

Several studies have shown that the activation of NF-κB may be associated with the induction of pancreatic β cell death [19,20]. Thus, due to our finding that GIP_HUMAN [22–51] promotes the nuclear translocation of NF-κB in INS-1E cells, we studied whether GIP_HUMAN [22–51] is involved in controlling INS-1E cell replication and cell death. As shown in Figure 3A,B, GIP_HUMAN [22–51] at all tested doses failed to modulate cell proliferation after 24 h (A) or 48 h (B). Furthermore, GIP_HUMAN [22–51] was not able to affect β cell death (Figure 3C).

## 4. Discussion

In the present study, we found that GIP_HUMAN [22–51] lowers insulin mRNA expression (*Ins1* and *Ins2*) in insulin-producing INS-1E cells as well as in freshly isolated rat pancreatic islets. Moreover, we report a potential mechanism through which GIP_HUMAN [22–51] may impact insulin mRNA expression in INS-1E cells. Due to a limited number of data, molecular mechanisms through which GIP_HUMAN [22–51] affects cellular functions has been poorly characterized so far. Nevertheless, a previous study reported that, in human aortic endothelial cells (HAoECs) or THP1-derived macrophages, GIP_HUMAN [22–51] induces translocation of NF-κB from cytoplasm to the nucleus [8]. Of note, NF-κB is a protein complex that, upon activation, is located in the nucleus where it modulates expression of genes mainly involved in inflammatory response as well as in cell death and proliferation [21]. Importantly, a previously published study showed that NF-κB may be involved in controlling insulin mRNA expression in pancreatic β cells. Amyot et al. reported that, in rat and human pancreatic islets, liposaccharides (LPS) suppress insulin mRNA expression via activation of NF-κB [18]. Therefore, since we found that GIP_HUMAN [22–51] reduces insulin mRNA expression, we evaluated its effect on NF-κB in INS-1E cells. Our data show that INS-1E cells exposed to GIP_HUMAN [22–51] had a higher immunoreactivity level of NF-κB (p65) in the nucleus. Furthermore, our results show that INS-1E cells treated with the NF-κB activation inhibitor IV ((E)-2-Fluoro-4′-methoxystilbene) [22] attenuated GIP_HUMAN [22–51] induced nuclear translocation of NF-κB. This effect was paralleled by the attenuation of *Ins2,* but not *Ins1,* mRNA expression. Therefore, our data suggest that the suppression of *Ins1* mRNA expression is rather independent on NF-κB activation.

This differential effect of *Ins1* and *Ins2* mRNA expression is known from previous studies that evaluated the transcriptional regulation of *Ins1* and *Ins2* mRNA expression [23,24]. Furthermore, it needs to be pointed out that, according to our knowledge, the direct mechanisms by which NF-κB modulates insulin gene expression have not been elucidated so far. However, Amyot at al. [18] found that suppression of insulin expression by GIP_HUMAN [22–51] is associated with downregulation of *Pdx1* mRNA expression, which is one of the major transcription factors involved in controlling insulin mRNA expression [25]. However, in our study, we did not find any changes in *Pdx1* mRNA levels in INS-1E cells (Supplementary Figure S1). Furthermore, according to our best knowledge, direct regulation of PDX1 by NF-κB in β cells has not been confirmed yet. Therefore, direct mechanisms linking NF-κB activation to the downregulation of *Ins1* and *Ins2* mRNA expression require further study. Transcriptome analysis of GIP_HUMAN [22–51]-treated β cells should be helpful for answering this question.

Furthermore, we observe that GIP_HUMAN [22–51] suppressed basal, but not glucose-induced, insulin secretion from INS-1E or from isolated rat pancreatic islets. In the present study, we did not evaluate the intracellular mechanism by which GIP_HUMAN [22–51] may suppress insulin secretion selectively at the low glucose concentration. It is well known that high levels of glucose-stimulated insulin secretion results from the closure of ATP-sensitive potassium channels, which leads to plasma membrane depolarization and the opening of voltage-sensitive calcium channels. Increases in intracellular calcium levels consequently cause insulin exocytosis [26]. In contrast to glucose-induced insulin secretion, insulin secretion at low glucose concentrations (usually referred to as ‘basal insulin release’) is not extensively characterized and is still a matter of debate [27]. Nevertheless, several studies have focused on the mechanisms and various factors that may trigger insulin secretion independently of glucose concentration. For example, it was found that basal glucose secretion may be modulated by metabolism of cholesterol and its plasma membrane cholesterol content in β cells islets [28]. Furthermore, in addition to potassium-dependent channels, pancreatic β cells express many other ion channels, such as members of the TRP superfamily channels [29,30]. These can be activated by peptide hormones and their presence may trigger a rise in intracellular calcium levels. Also, intracellular magnesium changes were implicated in controlling basal insulin secretion [31]. Electrophysiological studies would be useful for elucidating potential mechanisms by which GIP_HUMAN [22–51] may affect insulin secretion.

In summary, these results indicate that GIP_HUMAN [22–51] is not involved in controlling postprandial insulin exocytosis. Nevertheless, it needs to be pointed out that our experiments are limited to in vitro incubation of insulin-producing cell lines and isolated islets only. Therefore, the potential role of GIP_HUMAN [22–51] in controlling insulin secretion remains to be investigated in vivo or in a setting that allows for the preservation of islet microcirculation.

An additional finding in our study is that GIP_HUMAN [22–51] failed to affect insulin cell proliferation and viability. When discussing this point, it is worth noting that several research groups imply that NF-κB is involved in controlling pancreatic β cell death. For example, some studies indicate that in pancreatic β cells cytokines or pro-diabetic agents regulate cell viability and death via an NF-κB-dependent mechanism [19,20]. Moreover, activation of NF-κB in mice was linked to the development of diabetes and β cell failure [32]. Therefore, the activation of NF-κB by GIP_HUMAN [22–51] and the lack of its effect on cell death in our study might be surprising. Nevertheless, the role of NF-κB in regulating pancreatic β cell viability and death is a matter of speculation. For example, the inhibition of an NF-κB pathway in mice during embryogenesis is accompanied by reduced β cell mass [33]. Moreover, others have reported that the activation of NF-κB in β cells attenuates TNF-1α-induced β cell death [34,35]. Therefore, the consequences of Nf-kB activation in β cells are complex. Notably, Nf-kB activation via canonical and non-canonical pathways may cause different cellular responses [36]. Furthermore, it is likely that GIP_HUMAN [22–51] can activate other pathways, which can potentially counteract the proapoptotic effects of NF-κB.

In the present study, we did not evaluate the mechanisms through which GIP_HUMAN [22–51] activates Nf-kB. Nevertheless, Masaki et al. reported that GIP_HUMAN [22–51] causes the degradation of IκB-α [8], which is an important step leading to Nf-kB activation [37]. Therefore, we suggest that this mechanism may be responsible for the activation of Nf-kB in INS-1E cells. However, more data are needed to study this process in detail and to identify further upstream intracellular mechanisms.

In summary, we show here that GIP_HUMAN [22–51] may contribute to glucose metabolism via the regulation of insulin mRNA expression. GIP_HUMAN [22–51] is a potent proatherogenic peptide [8]. Atherosclerotic complications are common complications in patients with diabetes [38]. Therefore, the pharmacological blockade of GIP_HUMAN [22–51] signaling may be a promising tool for improving insulin production and may attenuate cardiovascular complications in diabetes and metabolic syndrome. Moreover, we observed that GIP_HUMAN [22–51] suppressed insulin mRNA expression and basal insulin secretion. Notably, it was reported that type 2 diabetic patients in the late phase of the disease have extremely low insulin production after decades, similar to type 1 diabetic patients [39]; however, they show paradoxically increased basal insulin secretion [27]. Therefore, in vivo experiments are needed to confirm our observations and to explore the role of GIP_HUMAN [22–51] in different stages and types of diabetes. Moreover, a putative receptor of GIP_HUMAN [22–51] has not been proposed yet, and this will be an additional obstacle to performing such studies.

In conclusion, we found that GIP_HUMAN [22–51] suppresses insulin expression and secretion in INS-1E cells and rat pancreatic islets, potentially through an NF-κB-dependent mechanism.

## Data Availability

The data presented in this study are available on request from the corresponding author.

## Supplementary Materials

The following supporting information can be downloaded at: https://www.mdpi.com/article/10.3390/genes14101910/s1, Figure S1. Effects of GIP_HUMAN [22–51] on *Pdx1* mRNA expression in INS-1E cells exposed to GIP_HUMAN [22–51] for 3 (A) and 24 h (B). Results are mean ± SEM, (*n* = 10–12).
