# Supplementary figures and images for: GIP_HUMAN [22–51] Peptide Encoded by the Glucose-Dependent Insulinotropic Polypeptide (GIP) Gene Suppresses Insulin Expression and Secretion in INS-1E Cells and Rat Pancreatic Islets
Source: Genes (Basel). 2023 Oct 5;14(10):1910. doi: 10.3390/genes14101910 (PMC10606481; doi:10.3390/genes14101910)

# Supplementary Figure 1

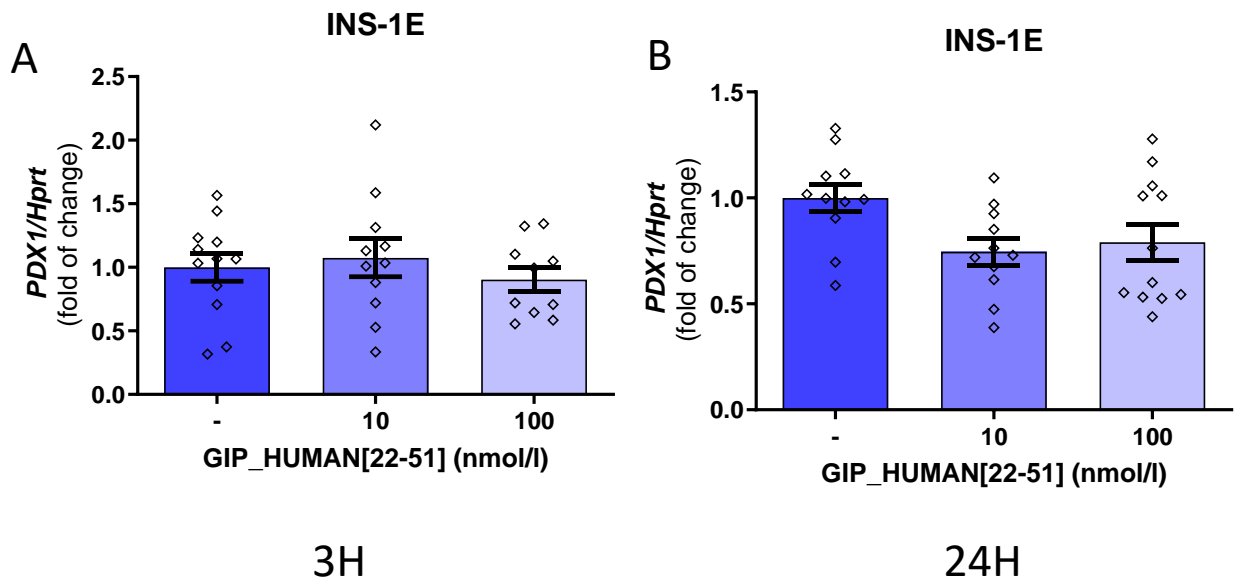

Supplement: Supplementary file 1 [file genes-14-01910-s001.zip › genes-2620153-supplementary.pdf]
